# Comparison of Caregiver-Reported Dietary Intake Methods in Zellweger Spectrum Disorder

**DOI:** 10.3390/nu17060989

**Published:** 2025-03-12

**Authors:** Mousumi Bose, Nancy L. von Thun, Adrian L. Kerrihard, Melisa L. Lopez, Chelsea I. Donlon, Alyssa K. Smolen, Nicole P. Fontes

**Affiliations:** Department of Nutrition and Food Studies, College for Community Health, Montclair State University, Montclair, NJ 07043, USA

**Keywords:** peroxisomal disorder, Zellweger spectrum disorder, rare disease, caregiver report, dietary record, nutrition requirement, Institute of Medicine, nutrients

## Abstract

**Background/Objectives**: Zellweger spectrum disorder (ZSD), a rare genetic disease characterized by defects in peroxisome biogenesis, results in dysfunction of all organ systems, including feeding difficulties, gastrointestinal bleeding, and reduced overall growth. Despite this nutritional impact, no published studies have assessed dietary intake in ZSD. The purpose of this study was to determine nutrient intake in individuals with ZSD or a related peroxisomal disorder using two methods of dietary assessment as provided by family caregivers. **Methods**: Family caregivers participated in multiple 24 h dietary recall interviews and completed 3-day food records for their child with ZSD or a related single-enzyme peroxisomal disorder over a 6-month period. **Results**: Twenty-one subjects (eleven orally fed and ten enterally fed), ranging from 1 to 33 years of age, were included in the study. Energy and nutrient intake as reported by dietary recall vs. 3-day food record were highly correlated for all nutrients (r^2^ = 0.998, *p* < 0.0001). Mean nutrient intakes for subjects generally achieved or exceeded DRI requirements, except for fiber (about 50% of DRI). **Conclusions**: These results show that dietary assessment is feasible in individuals with ZSD using caregiver input, regardless of feed modality, and that dietary intake is consistent across different methods of assessment. These findings may be applicable in dietary assessments for individuals with ZSD and similar genetic disorders and a methodological consideration in clinical interventions.

## 1. Introduction

Peroxisome biogenesis disorders (PBDs) are inherited disorders primarily caused by pathological variants in any of 14 different *PEX* genes. The *PEX* genes code for peroxins, proteins involved in peroxisome assembly and importation of peroxisomal matrix proteins [1]. PBDs are categorized into two groups: rhizomelic chondrodysplasia punctata [2] and Zellweger spectrum disorders [3].

Zellweger spectrum disorder (ZSD) is a group of autosomal recessive disorders with a reported incidence of ~1:50,000 births worldwide [2,3,4]. Incidence rates of ZSD, however, have varied dependent on geographical location. A founder disease-causing variant in a French-Canadian population resulted in a regional incidence rate of 1 in 12.191 births [5], while in Japan the estimated incidence of ZSD is reported to be around 1 in 500,000 births [6]. A more recent study using a bioinformatics approach reported an estimated incidence of 1 in 83,841 individuals [7].

The metabolic abnormalities of ZSD typically include elevated very long-chain fatty acids (VLCFAs) and defective bile acid synthesis, contributing to many of the clinical impacts of ZSD [8]. ZSD can range in severity from severe forms that result in a lifespan of 1–2 years of age to milder forms where patients can live into adulthood [4,9]. Affected individuals on the intermediate range of the spectrum often manifest low muscle tone, facial dysmorphism, impaired growth, sensory and neurological dysfunction, renal and endocrine insufficiency, skeletal abnormalities, and developmental delays [9,10,11,12,13,14,15,16,17,18,19,20,21]. ZSD can also affect nutritional status, including reduced overall growth [22] and feeding difficulties [23]. These characteristics of ZSD suggest that dietary intervention may be useful in managing some of the symptoms that affect some individuals with ZSD. Studies in other peroxisomal disorders have shown the benefit of dietary modification for symptom burden. A recent study reported that 96% of a population of patients with adult Refsum disease, a rare metabolic disease caused by a peroxisomal protein deficiency, followed a low-phytanic acid diet under the guidance of a dietitian [24]. Long-term dietary therapy with a low-phytanic acid diet has been shown to reduce symptom burden and hospital admissions in patients with adult Refsum disease [25]. A recent clinical trial observed the effects of antioxidant therapy in patients diagnosed with X-linked adrenoleukodystrophy, another peroxisomal protein deficiency disorder [26]. Previous clinical trials in X-ALD have also looked at the effects of Lorenzo’s oil, a mixture of oils including the fatty acids erucic and oleic acid, but have reported inconclusive results on its efficacy [27,28]. In ZSD patients, recent case studies have suggested that dietary intervention may be useful in the management of symptoms [29,30]. Moreover, in vitro studies in primary ZSD fibroblasts have suggested that various nutrients, such as amino acids, lipid compounds, and flavonoids, may restore peroxisomal function by acting as molecular chaperones to recover protein folding and importation defects [31,32]. However, intervention studies with dietary supplements in patients with ZSD have shown inconsistent results [33,34], and, to date, no studies in ZSD have assessed dietary intake as a measurement outcome in clinical studies for ZSD.

Due to the neurological impairment in moderate to severe ZSD, dietary assessments need to be modified from the traditional assessment methodology. In most cases, caregiver reports of dietary intake (as opposed to patient self-report) may be required due to speech difficulties and cognitive impairment. Additionally, many patients with ZSD require enteral feeding to address feeding difficulties [23]. To date, there are very few studies that have critically analyzed dietary assessment in children that require enteral feeding, despite studies that show that children on enteral feeding are at higher nutritional risk than orally fed children [35,36]. Moreover, many studies often exclude children who are enterally fed in dietary assessment studies conducted in neurologically impaired children [37]. Overall, there is little evidence-based guidance on conducting dietary assessments in children with cognitive delays and speech impairment, as well as in children who receive enteral feedings.

The primary purpose of this study was to determine the overall nutrient intake in subjects with ZSD or a related single-enzyme peroxisomal disorder, using the input of family caregivers. We conducted a series of dietary assessments (24 h dietary recalls and 3-day food records) at three time points to verify that these dietary assessments could provide reliable assessments of dietary intake in order to determine the overall nutrient intake in subjects with ZSD and related single-enzyme peroxisomal disorders. We partnered with the Global Foundation for Peroxisomal Disorders (GFPD; http://www.thegfpd.org; accessed on 21 January 2025), a patient advocacy group focused on ZSD and related peroxisomal disorders, to execute this study.

## 2. Materials and Methods

### 2.1. Recruitment

Approval for the study was granted by the Montclair State University Institutional Review Board (IRB-FY17-18-1053). All participants in this study were members of the Global Foundation for Peroxisomal Disorders (GFPD; https://www.thegfpd.org/). Study recruitment fliers were posted on the GFPD website and Facebook page to solicit enrollment. Individuals self-selected to participate. Inclusion criteria were being a family caregiver (parent, stepparent, or legal guardian) of a living child with a ZSD or a clinically similar single-enzyme peroxisome disorder (including acyl-CoA oxidase deficiency, alpha-methylacyl-CoA racemase deficiency, and D-bifunctional protein deficiency), one year of age or greater. All recruited participants had confirmed genetic and/or biochemical diagnosis of their child via caregiver report in the GFPD membership registry. Exclusion criteria were being a family caregiver of a child with ZSD or a related single-enzyme peroxisomal disorder who was receiving any breast milk, on parenteral nutrition, or less than 1 year of age at the time of the study. Prospective participants underwent a screener survey which included questions regarding demographics, feeding modality, overall diet, feeding time and frequency, support or modifications required during feeding, gastrointestinal symptoms, other symptoms related to the disorder (neurological and metabolic status), and current medications.

### 2.2. Study Period and Data Collection

The entire study was active from May 2020 until August 2023. Once informed consent was obtained and materials related to data collection (including a food scale, a fluid measuring cup, and a food model booklet with 2-D images to assist caregivers with estimation of portion size) were sent out to each participant, they were followed for about 6 months. Data were collected at the beginning of the study and 2 additional time points every 2 months. At each time point, a 24 h dietary recall and a 3-day food record for food, beverage, and supplement intake were completed for each participant’s child with a peroxisomal disorder and subsequently analyzed by a registered dietitian. Dietary recall interviews were conducted via Zoom video conference (version 6.0.0; San Jose, CA, USA) by a trained registered dietitian using Nutrition Data System for Research (NDSR 2020-2022; Nutrition Coordination Center, University of Minnesota, Minneapolis) software, which uses the automated multiple-pass method for 24 h dietary recall interviews [38]. This method asks the participant to give a general chronological overview of their child’s food consumption before inquiring in detail about serving sizes (using the food model booklet when applicable), preparation methods, brand names, and condiments. During each recall interview, participants were also asked to report on their child’s current height, weight, physical activity level, and symptoms related to feeding. Each recall interview concluded with the dietitian providing guidance to the participant on how to document their child’s food, beverage, and supplement intake for the 3-day food record. Participants were instructed to document intake (using the measurement instruments provided when applicable) for 2 weekdays and 1 weekend day for their child. Participants were asked to complete the 3-day food record within 2 weeks of having completed their recall interview for that time point. Energy, macronutrient, and micronutrient intake were determined for 24 h dietary recalls and 3-day food records using NDSR diet analysis software.

### 2.3. Data Analysis

#### 2.3.1. Determination of Orally vs. Enterally Fed Patients

Orally fed patients were determined to be those who received the majority (>50%) of their food intake through oral feeding. Enterally fed patients were determined to be those who received the majority (>50%) of their food and beverage intake through enteral tube feeding.

#### 2.3.2. Height and Weight Assessment

For patient subjects aged 20 years and under, the CDC (Centers for Disease Control) BMI-for-Age and Stature/Length-for-Age were used to determine the weight status and stature percentile of patients. For subjects above the age of 20 years, adult BMI charts were used to determine weight status, and the Stature-for-Age value at 20 years of age was used as the reference point for Stature-for-Age status for these patients [39].

#### 2.3.3. Energy and Nutrient Needs Assessment

All dietary recall and food record data were entered into the NDSR software. For each patient subject, the mean energy and nutrient intake for the 3 dietary recalls over the 6-month period were calculated. For the 3-day food records, the 3 days of recording were averaged for each measurement and then the means of those values over the 6-month period were calculated. Energy and nutrient data were entered into an Excel spreadsheet and expressed as a percentage of total intake compared to established Dietary Reference Intakes (DRIs) for each patient based on age and sex, as established for the Institute of Medicine (IOM) [40]. For total fat (lipid) intake, DRIs were determined to be achieving the lower limit of the Acceptable Macronutrient Distribution Range for the specific age and sex of each subject. For protein intake, given the small stature of most patients with ZSD or a related disorder, DRIs were determined to be based on g of protein per kg of body weight [41]. Estimated energy requirements were determined using the caregiver-reported sex, age, height, weight, and physical activity level of patients. Nutritional adequacy was defined as reaching 70% or greater of the DRI for the specific sex and life stage. Overnutrition was defined for sugar and saturated fat as greater than 10% of total caloric intake. For the other macronutrients and micronutrients, overnutrition was defined as caregiver-reported clinical signs of nutrient toxicity.

#### 2.3.4. Statistical Analysis

Correlations between the dietary recall and 3-day food record were determined within the assessments of energy and macronutrient intake. For this, the mean value of all time points was calculated for each participant within each energy and nutrient intake assessment. The coefficient of determination (R^2^) and probability value (*p*) were then determined by comparison of each participant’s mean dietary recall versus their mean 3-day food record for each energy and macronutrient intake (therefore utilizing 21 comparisons for each calculation). In consideration of possible biases due to small sample sizes, Fisher’s z-transformation was also performed and z’ values reported. Correlation data are presented in heat maps ranging from blue (lower correlation) to red (greater correlation).

Effect size for dietary recall versus 3-day food record was evaluated for each energy and nutrient intake value by calculation of mean differences with 95% confidence intervals and assessment of Hedges’ g.

Calculations were made using IBM SPSS Statistics 29.0 (Armonk, NY, USA: IBM Corp).

## 3. Results

### 3.1. Participant Demographics

A total of 23 caregivers of patients with ZSD or a related single-enzyme peroxisomal disorder consented to participate in the study to report on their child’s food, beverage, and supplement intake over the study period. Two were lost to follow-up, resulting in a total of twenty-one subjects who were included in the data analysis. One subject required parenteral nutrition during the course of the study and therefore was no longer eligible for the study after two dietary recalls and one food record were collected. The data collected from the two recalls and one food record from this subject were included in the analysis. Demographic data for subjects included in the study are reported in Table 1. Eight males and thirteen females, ranging from age 1 to 33 years, were represented by their caregivers in the study. The majority of the subjects were identified as White by their caregivers (*n* = 17), two subjects were identified as Black or African-African, and two subjects were identified as Hispanic or Latino/a/x. Fourteen out of twenty-one subjects were considered to be in the healthy weight to overweight range, and seven were considered to be in the underweight range. Five out of twenty-one subjects were considered to be under the 0th percentile for Height-for-Age, ten were in between the 0.1 to 10th percentile for Height-for-Age, three were in between the 11th to 50th percentile for Height-for-Age, and three subjects were above the 50th percentile for Height-for-Age. Eleven subjects received their primary nutrition via gastrostomy tube (n = 10) or by gastrojejunostomy tube (n = 1). The remaining 10 subjects received their nutrition via oral feeding. One subject was considered orally fed despite having a gastrostomy tube, as they only received fluids and medications through their gastrostomy tube.

### 3.2. Energy and Macronutrient Intake over the Study Period

Dietary macronutrient recall and food record data for subjects (patients) included in the study are reported in Table 2 and Table 3**.** For energy intake, reported as percent of total kilocalories (kcal) over established DRI, mean intake in subjects (expressed as mean ± standard deviation) was 90.3 ± 12.8% by dietary recall and 90.0 ± 9.2% by 3-day food record. For both recall and food record, 66.7% or greater of all subjects achieved 70% of their DRI for energy. For carbohydrate intake (reported as percent of total g over established DRI), mean intake in subjects was 128.3 ± 18.1% by dietary recall and 122.0 ± 11.2% by 3-day food record. For both recall and food record, 80.9% or greater of all subjects achieved 70% of their DRI for carbohydrate intake. For total fat (lipid) intake (reported as percent of total fat/kcal ratio over established DRI), mean intake in subjects was 154.2 ± 19.1% by dietary recall and 159.8 ± 8.3% by 3-day food record. For both recall and food record, all subjects achieved 70% of their DRI for total fat intake. For saturated fat intake (reported as percent of saturated fat/kcal ratio over established DRI), mean intake in subjects was 130.0 ± 20.3% by dietary recall and 135.8 ± 9.5% by 3-day food record. For both recall and food record, 85.7% or greater of all subjects achieved 70% of their DRI for saturated fat intake. For protein intake (reported as percent of total g/body weight ratio over established DRI), mean intake in subjects was 262.7 ± 40.5% by dietary recall and 255.7 ± 27.1% by 3-day food record. For both recall and food record, 95.2% or greater of all subjects achieved 70% of their DRI for protein intake. For fiber intake (reported as percent of total g over established DRI), mean intake in subjects was 55.5 ± 12.3% by dietary recall and 52.8 ± 9.1% by 3-day food record. For both recall and food record, 23.8% or less of all subjects achieved 70% of their DRI for fiber intake. For sugar intake (reported as percent of total sugar/kcal ratio over established DRI), mean intake in subjects was 214.2 ± 34.0% by dietary recall and 135.8 ± 9.5% by 3-day food record. For both recall and food record, 90.4% or greater of all subjects were over their DRI for sugar intake. A total of 52.3% or greater of all subjects were over 200% of their DRI for sugar intake.

### 3.3. Micronutrient Intake over the Study Period

Dietary micronutrient recall and food record data for subjects included in the study are reported in Table 4 and Table 5. For vitamin A intake, (reported as percent of total mcg of daily intake over established DRI), mean intake in subjects (expressed as mean ± standard deviation) was 622.5 ± 218.2% by dietary recall and 590.4 ± 343.4% by 3-day food record. For both recall and food record, 90.5% or greater of all subjects achieved 100% of their DRI for vitamin A. A total of 66.7% or greater of all subjects consumed over 200% of their DRI for vitamin A intake. For vitamin D intake, reported as percent of total mcg of daily intake over established DRI, mean intake in subjects was 241.2 ± 27.7% by dietary recall and 236.3 ± 35.4% by 3-day food record. For both recall and food record, 80.9% or greater of all subjects achieved 70% of their DRI for vitamin D intake. For vitamin E intake (reported as percent of total mcg of daily intake over established DRI), mean intake in subjects was 449.3 ± 67.7% by dietary recall and 415.7 ± 100.9% by 3-day food record. For both recall and food record, 85.7% or greater of all subjects achieved 70% of their DRI for vitamin E intake. For vitamin K intake (reported as percent of total mcg of daily intake ratio over established DRI), mean intake in subjects was 2624.8 ± 871.5% by dietary recall and 2909.0 ± 855.4% by 3-day food record. For both recall and food record, 90.4% of all subjects achieved 100% of their DRI for vitamin K intake, while 69.1% or greater of all subjects consumed over 200% of their DRI for vitamin K intake. For calcium intake (reported as percent of total mg of daily intake over established DRI), mean intake in subjects was 136.8 ± 30.0% by dietary recall and 128.0 ± 15.4% by 3-day food record. For both recall and food record, 85.7% or greater of all subjects achieved 70% of their DRI for calcium intake. For iron intake (reported as percent of total mg of daily intake over established DRI), mean intake in subjects was 336.7 ± 131.7% by dietary recall and 191.4 ± 85.4% by 3-day food record. For both recall and food record, 90.5% or less of all subjects achieved 70% of their DRI for iron intake. For sodium intake (reported as percent of total mg of daily intake over established DRI), mean intake in subjects was 138.3 ± 35.6% by dietary recall and 362.8 ± 109.2% by 3-day food record. For both recall and food record, 76.2% or greater of all subjects consumed over 70% of their DRI for sodium intake.

### 3.4. Correlation and Effect Size Across Dietary Assessment Methods

For all nutrients measured, Pearson’s r-squared values for 24 h recall and 3-day food record nutrient measurements indicated statistically significant correlations across assessment methods. Fisher’s z-transformation of r-squared values demonstrated similar trends, suggesting stability of variance despite the small sample size. Overall, saturated fat, sugar, and vitamin D intake were the most highly correlated factors across the assessment methods (r^2^ = 0.93, *p* < 0.00001; Table 5, Supplementary Figures S1 and S2). Vitamin A and sodium intake were the least correlated factors across the assessment methods (Table 6, Supplementary Figure S2).

The effect size, corresponding 95% confidence intervals, and Hedge’s g values for mean dietary intake measurements across dietary assessment methods are presented in Table 7. For all nutrients measured except sodium, iron, and vitamin E, the 95% confidence intervals for the mean differences overlap with 0 (indicating no statistically significant effect of assessment method).

## 4. Discussion

In the current study, we present the overall energy and nutrient intake of individuals with ZSD or a related single-enzyme peroxisomal disorder as determined by caregiver report. Additionally, we show the consistent reporting of dietary intake by caregivers on their affected children across two different methods of dietary assessment. To our knowledge, this is the first report of dietary intake in individuals with these disorders. Our study shows that, according to both 24 h recall and 3-day food records, patients, on average, were achieving or exceeding their daily requirements (as determined by DRI, established by the IOM) for energy, carbohydrate, protein, and total fat intake. Mean protein intake was greater than 2.5 times the daily requirements, with nearly all patients individually consuming 1.5 times the recommended amounts of protein daily or higher. These data suggest that patients should at least be consuming food and beverages to meet their estimated energy expenditure calorie requirement. Additionally, patients may need at least 1.5 times more protein than their DRI to maintain growth requirements. Although no overt kidney symptoms were reported in our study, kidney function has been recommended to be monitored in patients with ZSD [42] and should be followed in patients for both ZSD-related symptoms and the impacts of protein intake on the kidneys.

Our findings on adequate or higher than adequate macronutrient intake are in spite of the fact that a third of the patients in this study were underweight and the majority of patients were at or below the 10th percentile for stature. Failure to thrive is a common characteristic in ZSD and related peroxisomal disorders, and either increased caloric/macronutrient intake or enteral tube placement are often prescribed in these patients to address the failure-to-thrive diagnosis [42]. The findings of our study suggest that either calorie and nutrient requirements are higher in patients with ZSD compared to the age-matched reference values established by the IOM, or that there is altered growth in individuals with ZSD. Given the low muscle tone present in ZSD and related disorders and its general impact of reduced mobility [23], energy expenditure is likely not higher in these patients. However, bile acid synthesis defects are a common occurrence in moderate to severe ZSD [43], which may affect nutrient absorption, particularly the absorption of fat and fat-soluble vitamins. Therefore, calorie and nutrient requirements may be higher in patients with ZSD and related disorders due to nutrient malabsorption. Future studies will need to determine whether or not nutrient malabsorption is a significant contributor to growth issues in this patient population.

Regarding the possibility of altered growth in ZSD and related disorders, bone mineral density is often reduced in moderate to severe ZSD [10], and a 2017 paper published a disease-specific growth chart for rhizomelic chondrodysplasia punctata (RCDP), another peroxisome biogenesis disorder, based on decreased growth potential in these patients [44]. Growth monitoring in ZSD is recommended periodically in patients, and a 2018 study that developed a tool to measure disease severity in patients with ZSD included growth as one of its measurement domains [45]. Taken together with our data, there may be a need for a condition-specific growth chart for ZSD and related disorders.

Fiber was the only nutrient whose consumption was consistently lower in our patients compared to the recommended intakes. Inadequate fiber intake is a common public health problem among all children and adults in the United States [46]. Similarly, sugar consumption was consistently above the recommended intake levels for almost all subjects in this study, which aligns with sugar consumption trends among children in the United States [47,48]. Strategies to address these issues in individuals with ZSD or a related peroxisomal disorder should follow general recommendations to increase fiber intake and decrease sugar intake. However, on an individual basis, it is important that families discuss any considerable dietary changes with their medical professionals before implementation to avoid any potential adverse effects.

Mean intake of micronutrients, as determined by recall or food record, was also adequate or above DRI requirements among our subjects. Mean fat-soluble vitamin intake ranged from approximately 2.5 times over the DRI (for vitamin D) to over 200 times the DRI (for vitamin K). Malabsorption of fat-soluble vitamins due to bile acid synthesis defects has been characterized in ZSD, and fat-soluble vitamin supplementation is recommended to severe and moderate ZSD patients [42]. Mean vitamin K intake was particularly high compared to the other fat-soluble vitamins; this was likely meant to reduce the risk of coagulopathy often observed in moderate to severe ZSD due to liver dysfunction and vitamin K malabsorption [13]. The majority of the patients studied were taking additional vitamin K supplements above and beyond the standard fat-soluble supplementation that is recommended for patients with moderate to severe ZSD [42].

For all nutrients studied, we observed statistically significant correlations between caregiver reports of dietary intake by 24 h dietary recall vs. 3-day food recall in patients. This shows that the caregiver reporting of dietary intake was consistent across the methodologies in this study, suggesting that caregivers of these patients can provide an accurate assessment of food intake, and, consequently, these data can be used as measurable outcomes in clinical practice and research. Our data serve as a proof of concept to conduct possible dietary intervention studies with nutrients that may reduce symptom burden in ZSD. As an example, docosahexaenoic acid (DHA) is an essential polyunsaturated fatty acid (C22:6) important for neuronal cell communication as well as retinal function [49]. Peroxisomes are the primary sites of DHA metabolism [50], and individuals with ZSD have been shown to have reduced levels of DHA in brain tissue [51]. Intervention studies with DHA supplementation to date, however, have yielded inconsistent results regarding its effectiveness in patients with ZSD [33,34,52]. It should be noted that none of these studies included a full dietary assessment of subjects with respect to food, beverage, and supplement intake. Depending on the DHA supplement dose used in these studies, the overall dietary contribution of DHA may have been a confounding variable in these studies. Taken together, there may be a rationale for more controlled clinical trials with DHA supplementation that include a comprehensive dietary assessment of DHA intake as well as other nutrients.

Additionally, there is a growing interest in the role of phytonutrients such as flavonoids from the diet and in supplement form in the management of neurodegenerative diseases in adults [53]. An in vitro study of fibroblasts derived from ZSD patients showed that diosmetin and other related flavanols restored peroxisomal activity [54]. These data suggest that dietary supplementation of flavanols may be useful in the management of symptoms of ZSD by restoring peroxisomal activity; a dietary intervention study with dietary assessment outcomes would be warranted in this case.

The correlation between methodologies was stronger for macronutrient intake compared to micronutrient intake. Additionally, the effect size was significant across 24 h recall and 3-day food records for three micronutrients, including vitamin E, iron, and sodium. This may be due to the fact that the majority of caregivers considered dietary supplements as their child’s primary source of micronutrient intake as supplements and may have been less consistent with supplement intake compared to actual food and beverage intake. Indeed, in our supplement assessment, some caregivers reported their child missing days of taking their dietary supplements, which may have resulted in differences in intake across the two assessment methods.

Between the two dietary assessment methodologies, the 3-day food record approach has been associated with greater respondent burden compared to the 24 h recall method [55]. Considering the heavy burden of responsibilities that caregivers of patients with ZSD experience [56], the 24 h dietary recall may be the more appropriate methodology to conduct dietary assessments in families affected by ZSD or disorders with a similar burden.

Although this study presents new findings on dietary intake of individuals diagnosed with ZSD or a related single-enzyme peroxisomal disorder, there are some limitations to the research. First, given the rarity of these disorders, our sample size was small, and our subjects were spread out across the United States, Canada, and the United Kingdom. Therefore, we were unable to conduct in-person assessments. As a result, we relied on caregiver reporting of height and weight based on their child’s last physician appointment. This may have played a role in some inaccuracies in height and weight. Additionally, although both recall and food records were highly correlated across nutrient intake, suggesting that caregivers can report on their child’s food intake consistently across the two methodologies, we cannot definitively say that these reports were an accurate reflection of actual intake, as both sets were caregiver reports and were not compared against a more absolute measure, such as biomarkers of nutrient levels in blood samples. Future studies will need to compare dietary intake with laboratory data to confirm the accuracy of nutrient intake methods.

It is possible that the strong correlations in nutrient intake between the different dietary assessment methods are due to the fact that many enterally fed patients likely receive the same formula and volume every day, which would lend to its consistency across dietary assessment methods. Conversely, some of the differences observed in micronutrient intake across assessment methods may also be partially attributable to feeding modality. Future studies will tease out nutrient intake and correlations between assessment methods in orally and enterally fed patients to see if there are differences in outcomes across these different feeding modalities.

Our study used the IOM DRIs as the reference values for energy and nutrient intake due to their accounting for age in recommended intakes. Some research has suggested that IOM estimated energy requirements overestimate caloric needs, although this overestimation has been generally observed in obese subjects [57]. As our subjects are not obese and are potentially at risk for undernutrition, our observation of adequate or higher than adequate intake among our subjects against IOM DRIs further supports that our subjects are meeting their caloric and nutrient needs.

## 5. Conclusions

Our study presents the first report on the caloric and nutrient intake of patients diagnosed with ZSD and related single-enzyme peroxisomal disorders. Moreover, our data show that dietary assessment via caregiver report is feasible for clinical and research purposes in individuals with ZSD or in similar neurological disorders. These data can be useful in the development of dietary interventions that may translate into clearer and more standardized dietary recommendations for ZSD and the broader rare-disease community.

## Figures and Tables

**Table 1:** Subject demographic data.

Subj	Diagnosis	Age (y)	Sex	Feeding Mode ^	Reported Symptoms	Race/Ethnicity	BMI Weight Status *
1	ZSD	1.0	F	O	C, Ge	White	Healthy weight
2	ZSD	2.0	F	O	Ch	White	Healthy weight
3	ZSD	4.9	M	O	D	Hispanic/Latinx	Healthy weight
4	ZSD	7.0	M	O	D, R, V	Black	Overweight
5	ZSD	7.0	F	O	C	Hispanic/Latinx	Healthy weight
6	ZSD	7.2	F	O	C	White	Underweight
7	ZSD	8.0	F	O	none	White	Healthy weight
8	ZSD	10.0	M	O	C, R	White	Underweight
9	ZSD	21.0	M	O	F	White	Healthy weight
10	DBPD	23.0	F	O	C	White	Healthy weight
11	ZSD	33.0	F	O	F, Fr	White	Healthy weight
12	ZSD	1.1	M	E	C, GI, R, V	White	Underweight
13	ZSD	2.0	F	E	C	White	Healthy weight
14	ZSD	3.0	F	E	GI	White	Healthy weight
15	ZSD	4.3	F	E	C, GI, V	White	Healthy weight
16	ZSD	5.0	M	E	V	White	Healthy weight
17	ZSD	7.0	M	E	None	White	Overweight
18	ZSD	10.0	M	E	None	White	Healthy weight
19	ZSD	12.0	F	E	None	White	Healthy weight
20	ZSD	13.0	F	E	C, Ge	Black	Underweight
21	ZSD	20.0	F	E	C, Ge	White	Underweight

* For subjects aged 20 years and under, the CDC (Centers for Disease Control) BMI-for-Age and Stature/Length-for-Age were used to determine weight status and stature percentile of subjects. For subjects above the age of 20 years, adult BMI charts were used to determine weight status, and Stature-for-Age value at 20 years of age was used as the reference point for Stature-for-Age status for these subjects. ^ For orally fed subjects, about 95% or more of their food intake was through oral feeding. One subject who was considered an orally fed subject had a gastrostomy tube but only received fluids and medications through their gastrostomy tube. For enterally fed subjects, all subjects received 95% or more of their food and beverage intake through their gastrostomy tube; one subject received all of their nutrition through a gastrojejunostomy tube. Abbreviations: C = constipation, Ch = chewing difficulties, D = diarrhea, E = enterally fed, F = falls, Fr = fractures, Ge = gastroesophageal reflux disease, GI = gastrointestinal bleed, O = orally fed, V = vomiting.

**Table 2:** Macronutrient intake (as a percentage of DRI) of individual subjects determined by caregiver-reported 24 h dietary recall.

Subj	Age (y)	Sex	Feeding Mode	Energy	Carbohydrates	Proteins	Fat	Saturated Fat	Fiber	Sugar
1	1.0	F	O	128.0 ± 6.4	90.9 ± 11.7	235.0 ± 8.5	145.7 ± 16.3	144.1 ± 21.2	38.2 ± 4.0	206.9 ± 24.6
2	2.0	F	O	131.0 ± 18.3	91.5 ± 4.8	345.4 ± 114.4	170.1 ± 47.9	198.0 ± 37.3	40.7 ± 10.9	236.2 ± 86.1
3	4.9	M	O	87.0 ± 16.6	157.0 ± 56.4	390.5 ± 87.9	154.4 ± 45.6	82.6 ± 30.8	127.5 ± 48.7	110.3 ± 30.1
4	7.0	M	O	85.0 ± 9.1	214.3 ± 14.0	155.8 ± 15.7	152.4 ± 5.9	65.4 ± 4.4	21.5 ± 2.0	115.9 ± 6.1
5	7.0	F	O	71.2 ± 11.7	96.5 ± 13.4	147.3 ± 20.0	92.4 ± 16.5	87.9 ± 24.9	63.1 ± 21.8	214.5 ± 58.6
6	7.2	F	O	83.6 ± 20.9	151.7 ± 32.3	384.7 ± 75.5	160.3 ± 12.5	181.5 ± 27.2	74.7 ± 22.1	182.7 ± 11.8
7	8.0	F	O	83.0 ± 16.6	199.1 ± 63.9	294.4 ± 56.0	85.7 ± 35.1	74.3 ± 16.1	47.7 ± 10.3	378.4 ± 59.6
8	10.0	M	O	87.5 ± 22.2	135.1 ± 24.6	220.1 ± 86.9	176.0 ± 8.5	98.1 ± 29.0	50.3 ± 10.6	236.1 ± 72.0
9	21.0	M	O	83.7 ± 20.6	292.4 ± 50.6	202.6 ± 7.2	95.1 ± 8.8	52.9 ± 4.0	70.1 ± 13.4	455.3 ± 13.8
10	23.0	F	O	49.0 ± 10.5	108.2 ± 29.2	159.3 ± 90.8	153.8 ± 5.4	121.2 ± 29.7	50.3 ± 21.5	166.9 ± 96.4
11	33.0	F	O	96.4 ± 18.1	199.0 ± 5.3	232.5 ± 39.5	149.4 ± 18.7	126.0 ± 6.8	40.6 ± 9.9	283.5 ± 67.8
12	1.1	M	E	109.8 ± 12.7	66.5 ± 4.2	239.0 ± 40.8	147.2 ± 12.9	133.2 ± 37.5	41.1 ± 12.8	214.5 ± 58.6
13	2.0	F	E	88.7 ± 10.1	51.4 ± 2.7	188.9 ± 10.9	138.5 ± 6.7	125.5 ± 43.9	22.4 ± 22.4	152.2 ± 67.0
14	3.0	F	E	63.0 ± 4.8	74.3 ± 2.7	246.2 ± 13.0	105.1 ± 26.7	206.1 ± 1.1	37.5 ± 2.2	119.2 ± 7.2
15	4.3	F	E	72.0 ± 0.9	17.0 ± 0.4	154.9 ± 0.0	356.4 ± 0.7	356.2 ± 0.7	52.6 ± 0.0	26.1 ± 1.7
16	5.0	M	E	135.0 ± 11.3	198.2 ± 26.2	503.7 ± 63.4	184.2 ± 28.2	85.0 ± 56.4	132.6 ± 19.7	179.0 ± 17.6
17	7.0	M	E	80.0 ± 18.2	87.3 ± 1.5	197.5 ± 2.0	163.1 ± 29.3	167.7 ± 0.9	37.7 ± 0.4	256.7 ± 2.0
18	10.0	M	E	72.3 ± 5.5	140.9 ± 12.8	268.6 ± 64.0	203.5 ± 9.8	132.3 ± 5.8	64.0 ± 15.5	139.9 ± 31.3
19	12.0	F	E	105.2 ± 17.2	157.3 ± 1.5	213.7 ± 3.3	137.4 ± 10.8	110.3 ± 0.3	37.2 ± 0.1	290.5 ± 0.3
20	13.0	F	E	147.6 ± 2.7	96.0 ± 0.3	680.0 ± 27.1	154.4 ± 1.2	100.3 ± 18.7	96.1 ± 0.0	79.7 ± 0.1
21	20.0	F	E	36.4 ± 13.0	69.7 ± 21.9	56.8 ± 23.1	114.6 ± 54.4	82.7 ± 28.7	19.3 ± 10.0	535.2 ± 97.4
**Total Nutrient Mean**				**90.3 ± 12.8**	**128.3 ± 18.1**	**262.7 ± 40.5**	**154.2 ± 19.1**	**130.0 ± 20.3**	**55.5 ± 12.3**	**214.2 ± 34.0**

The 24 h dietary recall interviews were conducted via Zoom conference by a trained registered dietitian using Nutrition Data System for Research software. Energy and macronutrient intake were determined as a percentage of total intake compared to established Dietary Reference Intakes (DRIs) for each subject (Subj) based on age and sex, as established for the Institute of Medicine [38]. Data are reported as mean percentage values for 3 dietary recalls over a 6-month period ± standard deviations of the means. Abbreviations for feeding mode: O = orally fed, E = enterally fed.

**Table 3:** Macronutrient intake (as a percentage of DRI) of individual subjects determined by caregiver-reported 3-day dietary food record.

Subj	Age (y)	Sex	Feeding Mode	Energy	Carbohydrates	Proteins	Fat	Saturated Fat	Fiber	Sugar
1	1.0	F	O	143.4 ± 20.3	114.5 ± 21.3	207.5 ± 18.0	116.7 ± 17.2	128.4 ± 24.0	52.6 ± 16.5	249.6 ± 48.9
2	2.0	F	O	104.8 ± 2.0	70.0 ± 1.8	312.1 ± 27.8	146.1 ± 1.3	204.9 ± 4.6	25.1 ± 4.9	227.4 ± 25.9
3	4.9	M	O	67.8 ± 6.7	124.5 ± 15.5	330.0 ± 95.4	131.8 ± 14.5	82.9 ± 8.4	86.1 ± 10.9	116.2 ± 6.9
4	7.0	M	O	86.6 ± 19.3	220.7 ± 44.8	155.8 ± 24.1	139.8 ± 5.4	59.0 ± 0.4	21.4 ± 1.9	111.6 ± 14.5
5	7.0	F	O	84.1 ± 11.5	112.1 ± 14.0	169.8 ± 24.1	91.8 ± 5.1	117.8 ± 1.0	44.4 ± 17.5	210.9 ± 7.0
6	7.2	F	O	64.6 ± 6.4	119.7 ± 14.7	296.9 ± 33.2	159.2 ± 8.5	169.8 ± 14.6	54.8 ± 7.9	206.2 ± 41.2
7	8.0	F	O	66.5 ± 10.7	143.1 ± 6.4	212.8 ± 47.7	117.1 ± 20.4	97.2 ± 27.0	39.0 ± 5.0	326.6 ± 47.9
8	10.0	M	O	74.2 ± 8.7	97.3 ± 7.6	222.1 ± 28.3	215.5 ± 6.4	145.0 ± 10.5	32.6 ± 5.4	172.9 ± 12.8
9	21.0	M	O	91.4 ± 5.5	297.3 ± 13.4	256.0 ± 58.7	100.6 ± 12.3	55.7 ± 2.1	84.3 ± 25.4	377.4 ± 61.8
10	23.0	F	O	55.3 ± 4.6	115.2 ± 9.3	168.7 ± 24.6	171.1 ± 4.7	152.0 ± 25.2	52.1 ± 10.3	139.2 ± 42.4
11	33.0	F	O	77.8 ± 8.1	158.9 ± 10.6	209.7 ± 37.9	151.8 ± 6.2	121.5 ± 5.3	38.2 ± 6.4	276.2 ± 19.0
12	1.1	M	E	126.8 ± 11.1	67.6 ± 4.9	249.8 ± 14.6	155.0 ± 13.5	125.9 ± 35.0	55.4 ± 133.5	127.6 ± 20.6
13	2.0	F	E	98.8 ± 0.0	54.0 ± 0.0	199.8 ± 0.0	145.2 ± 0.0	169.3 ± 0.0	0.0 ± 0.0	136.7 ± 0.0
14	3.0	F	E	64.0 ± 2.8	76.0 ± 6.7	254.5 ± 18.9	122.8 ± 1.7	205.8 ± 0.0	38.7 ± 2.6	120.1 ± 4.3
15	4.3	F	E	72.6 ± 0.2	17.2 ± 0.4	154.9 ± 0.0	356.4 ± 0.7	356.2 ± 0.7	52.6 ± 0.0	26.4 ± 1.5
16	5.0	M	E	112.8 ± 7.0	168.7 ± 11.1	410.7 ± 11.0	168.9 ± 14.1	62.7 ± 8.4	111.9 ± 0.5	201.6 ± 23.2
17	7.0	M	E	81.1 ± 17.4	89.3 ± 0.0	201.4 ± 1.3	182.9 ± 0.0	166.5 ± 0.0	38.2 ± 0.0	259.4 ± 0.0
18	10.0	M	E	66.1 ± 2.9	125.2 ± 9.1	275.8 ± 21.8	215.8 ± 9.1	138.1 ± 5.0	56.0 ± 6.0	151.6 ± 8.0
19	12.0	F	E	103.5 ± 15.0	155.1 ± 1.9	210.5 ± 6.1	145.1 ± 0.1	110.5 ± 0.6	36.5 ± 0.9	289.6 ± 1.1
20	13.0	F	E	199.5 ± 19.8	155.6 ± 25.0	772.5 ± 27.4	149.8 ± 12.0	75.8 ± 11.2	158.5 ± 18.6	75.6 ± 6.8
21	20.0	F	E	48.4 ± 13.1	80.2 ± 16.5	99.4 ± 34.7	173.4 ± 21.3	106.1 ± 12.4	30.4 ± 17.6	401.4 ± 129.2
**Total Nutrient Mean**				**90.0 ± 9.2**	**122.0 ± 11.2**	**255.7 ± 27.1**	**159.8 ± 8.3**	**135.8 ± 9.4**	**52.8 ± 9.1**	**200.2 ± 30.2**

For the 3-day dietary food record, caregivers (participants) were instructed to document food, beverage, and supplement intake for 2 weekdays and 1 weekend day for their child (subject). Participants were asked to complete the 3-day food record within 2 weeks of having completed their recall interview for that time point. Energy and macronutrient intake were determined as a percentage of total intake compared to established Dietary Reference Intakes (DRIs) for each subject (Subj) based on age and sex, as established for the Institute of Medicine [38]. Data are reported as mean percentage values for 3 dietary food records over a 6-month period ± standard deviations of the means. Abbreviations for feeding mode: O = orally fed, E = enterally fed.

**Table 4:** Individual micronutrient intake (as a percentage of DRI) determined by caregiver-reported dietary recall.

Subj	Age (y)	Sex	Feeding Mode	Vitamin A	Vitamin D	Vitamin E	Vitamin K	Calcium	Iron	Sodium
1	1.0	F	O	644.7 ± 533.6	226.4 ± 28.2	798.8 ± 62.0	895.7 ± 463.5	235.9 ± 122.1	101.3 ± 40.5	327.5 ± 178.0
2	2.0	F	O	253.2 ± 91.6	73.9 ± 42.1	83.8 ± 58.4	8417.8 ± 14.1	151.6 ± 63.0	83.6 ± 6.6	213.0 ± 60.6
3	4.9	M	O	924.9 ± 352.8	149.6 ± 9.5	254.4 ± 5.2	228.3 ± 49.6	111.1 ± 31.1	181.5 ± 8.2	204.1 ± 45.4
4	7.0	M	O	214.6 ± 24.6	207.0 ± 25.0	341.6 ± 38.0	279.3 ± 31.4	195.2 ± 21.6	235.0 ± 36.3	162.9 ± 3.3
5	7.0	F	O	287.2 ± 329.5	20.1 ± 13.9	52.3 ± 13.7	72.7 ± 36.9	70.9 ± 28.6	108.6 ± 32.7	119.9 ± 49.5
6	7.2	F	O	2380.5 ± 1474.6	28.1 ± 7.8	84.1 ± 19.4	253.5 ± 58.7	159.2 ± 41.4	70.0 ± 1.4	222.5 ± 77.6
7	8.0	F	O	1517.0 ± 86.5	452.2 ± 66.4	999.9 ± 17.5	1868.0 ± 21.3	196.4 ± 61.6	405.7 ± 238.6	291.0 ± 56.4
8	10.0	M	O	215.1 ± 124.9	165.1 ± 12.1	231.6 ± 64.4	8390.8 ± 43.6	60.7 ± 26.5	610.0 ± 212.3	99.5 ± 37.0
9	21.0	M	O	170.4 ± 31.8	446.8 ± 5.0	212.4 ± 24.8	4296.7 ± 52.4	348.4 ± 66.2	391.2 ± 30.0	126.9 ± 33.1
10	23.0	F	O	645.6 ± 688.2	291.2 ± 51.9	42.4 ± 5.1	129.6 ± 80.8	94.0 ± 19.0	65.0 ± 8.8	147.7 ± 50.9
11	33.0	F	O	130.8 ± 46.7	962.7 ± 9.1	166.7 ± 15.1	137.8 ± 10.0	158.6 ± 47.3	71.1 ± 4.7	197.2 ± 19.7
12	1.1	M	E	741.6 ± 438.4	157.7 ± 40.0	575.6 ± 177.8	132.2 ± 14.9	104.7 ± 29.7	141.9 ± 1.6	43.0 ± 4.5
13	2.0	F	E	164.6 ± 17.8	123.3 ± 10.0	118.5 ± 12.2	251.3 ± 33.7	94.6 ± 17.0	127.2 ± 42.9	47.9 ± 9.2
14	3.0	F	E	408.1 ± 232.5	317.1 ± 87.8	1268.0 ± 482.6	11,336.9 ± 7823.7	111.7 ± 5.9	2140.8 ± 1586.6	54.8 ± 2.6
15	4.3	F	E	134.8 ± 0.0	208.9 ± 31.4	366.9 ± 25.3	160.1 ± 25.7	101.1 ± 3.3	652.1 ± 329.9	70.8 ± 0.3
16	5.0	M	E	607.3 ± 76.5	250.4 ± 13.6	1314.0 ± 84.4	12,305.6 ± 8574.0	228.1 ± 13.0	246.7 ± 90.6	165.8 ± 26.5
17	7.0	M	E	1162.9 ± 2.9	256.1 ± 6.0	1170.4 ± 3.2	3496.1 ± 205.9	78.6 ± 2.3	610.7 ± 14.5	53.1 ± 1.3
18	10.0	M	E	91.0 ± 11.1	200.5 ± 105.8	402.3 ± 286.4	674.7 ± 755.7	113.0 ± 9.7	261.9 ± 15.4	137.5 ± 76.5
19	12.0	F	E	755.4 ± 1.1	356.8 ± 1.3	825.2 ± 2.1	1456.5 ± 1.0	143.8 ± 1.2	254.1 ± 2.4	93.4 ± 0.7
20	13.0	F	E	1526.7 ± 7.4	127.1 ± 1.6	96.1 ± 4.5	316.3 ± 0.6	67.2 ± 1.3	274.3 ± 47.2	50.5 ± 2.4
21	20.0	F	E	96.2 ± 10.3	43.9 ± 14.2	30.2 ± 19.4	21.6 ± 5.1	47.3 ± 17.8	38.6 ± 14.4	74.5 ± 12.1
**Total Nutrient Mean**				**622.5 ± 218.2**	**241.2 ± 27.7**	**449.3 ± 67.7**	**2624.8 ± 871.5**	**136.8 ± 30.0**	**336.7 ± 131.7**	**138.3 ± 35.6**

The 24 h dietary recall interviews were conducted via Zoom conference by a trained registered dietitian using Nutrition Data System for Research software. Micronutrient intake was determined as a percentage of total intake compared to established Dietary Reference Intakes (DRIs) for each subject (Subj) based on age and sex, as established for the Institute of Medicine [38]. Data are reported as mean percentage values for 3 dietary recalls over a 6-month period ± standard deviations of the means. Abbreviations for feeding mode: O = orally fed, E = enterally fed.

**Table 5:** Individual micronutrient intake (as a percentage of DRI) determined by caregiver-reported dietary food record.

Subj	Age (y)	Sex	Feeding Mode	Vitamin A	Vitamin D	Vitamin E	Vitamin K	Calcium	Iron	Sodium
1	1.0	F	O	1314.5 ± 1432.0	198.9 ± 63.2	844.2 ± 258.2	159.4 ± 19.3	100.9 ± 28.3	125.2 ± 33.1	200.3 ± 28.3
2	2.0	F	O	188.4 ± 107.1	73.1 ± 21.5	71.9 ± 43.7	8387.1 ± 6.6	159.3 ± 17.6	73.8 ± 1.9	176.6 ± 11.9
3	4.9	M	O	217.3 ± 181.3	163.1 ± 16.9	214.3 ± 26.1	173.0 ± 18.0	132.2 ± 37.0	192.2 ± 8.0	240.9 ± 45.2
4	7.0	M	O	201.1 ± 60.7	212.9 ± 75.6	278.1 ± 87.2	267.4 ± 78.4	187.6 ± 57.0	320.0 ± 87.3	247.6 ± 21.2
5	7.0	F	O	499.9 ± 461.1	40.2 ± 9.2	44.8 ± 18.2	92.6 ± 102.0	74.7 ± 5.9	120.3 ± 42.4	158.7 ± 20.7
6	7.2	F	O	1051.8 ± 353.8	29.2 ± 5.6	70.2 ± 9.0	203.8 ± 38.2	113.1 ± 21.8	76.7 ± 10.0	208.3 ± 13.7
7	8.0	F	O	1109.4 ± 605.8	375.5 ± 18.7	752.7 ± 346.9	1122.2 ± 677.4	132.5 ± 33.7	558.8 ± 54.9	1276.7 ± 1478.0
8	10.0	M	O	192.9 ± 114.5	179.0 ± 26.5	220.5 ± 83.8	5869.0 ± 3619.5	75.1 ± 9.9	613.7 ± 217.6	156.5 ± 21.0
9	21.0	M	O	168.5 ± 59.7	465.9 ± 3.2	178.7 ± 54.6	4345.7 ± 88.8	335.6 ± 12.3	423.3 ± 18.2	153.1 ± 18.5
10	23.0	F	O	414.4 ± 87.3	320.4 ± 2.7	40.6 ± 1.7	172.0 ± 22.3	103.2 ± 15.7	66.0 ± 7.5	209.3 ± 35.6
11	33.0	F	O	83.1 ± 33.5	706.5 ± 362.5	118.8 ± 61.4	141.2 ± 4.3	142.9 ± 16.3	97.0 ± 48.2	194.9 ± 10.9
12	1.1	M	E	697.0 ± 415.3	192.1 ± 15.6	697.4 ± 34.1	3092.4 ± 4055.0	115.9 ± 8.5	183.0 ± 59.3	64.4 ± 11.4
13	2.0	F	E	54.7 ± 0.0	133.2 ± 0.0	154.5 ± 0.0	285.0 ± 0.0	111.6 ± 0.0	170.0 ± 0.0	38.7 ± 0.0
14	3.0	F	E	649.4 ± 806.7	389.7 ± 49.4	1452.2 ± 200.6	12,345.0 ± 8690.4	114.7 ± 8.2	2135.8 ± 1625.6	68.3 ± 14.0
15	4.3	F	E	134.8 ± 0.0	253.3 ± 0.0	402.6 ± 0.0	196.4 ± 0.0	105.7 ± 0.1	885.5 ± 0.1	85.2 ± 0.2
16	5.0	M	E	1783.4 ± 1115.7	252.1 ± 8.6	898.1 ± 391.6	18,338.4 ± 3.1	214.4 ± 5.3	155.3 ± 4.3	121.8 ± 3.4
17	7.0	M	E	1618.2 ± 587.2	255.2 ± 0.0	1024.6 ± 189.0	3353.9 ± 0.0	81.7 ± 0.0	592.0 ± 0.0	54.7 ± 0.0
18	10.0	M	E	111.2 ± 58.3	200.2 ± 50.6	338.1 ± 166.9	755.8 ± 434.7	123.4 ± 36.1	219.5 ± 29.2	104.1 ± 20.1
19	12.0	F	E	830.4 ± 284.2	354.6 ± 1.8	751.4 ± 103.5	1454.3 ± 2.3	141.3 ± 2.2	250.4 ± 3.2	91.8 ± 1.3
20	13.0	F	E	882.3 ± 339.2	124.6 ± 1.4	116.2 ± 14.3	308.3 ± 89.0	69.7 ± 2.4	312.3 ± 33.4	71.0 ± 11.5
21	20.0	F	E	196.7 ± 107.8	42.9 ± 11.0	59.5 ± 28.9	25.4 ± 14.7	51.7 ± 6.0	48.1 ± 10.0	97.2 ± 26.9
**Total Nutrient Mean**				**590.4 ± 343.4**	**236.3 ± 35.4**	**415.7 ± 100.9**	**2909.0 ± 855.4**	**128.0 ± 15.4**	**191.4 ± 85.4**	**362.8 ± 109.2**

For the 3-day dietary food record, caregivers (participants) were instructed to document food, beverage, and supplement intake for 2 weekdays and 1 weekend day for their child (subject). Participants were asked to complete the 3-day food record within 2 weeks of having completed their recall interview for that time point. Energy and macronutrient intake were determined as a percentage of total intake compared to established Dietary Reference Intakes (DRI) for each subject (Subj) based on age and sex, as established for the Institute of Medicine [38]. Data are reported as mean percentage values for 3 dietary food records over a 6-month period ± standard deviations of the means. Abbreviations for feeding mode: O = orally fed, E = enterally fed.

**Table 6:** Correlations between nutrient intakes as reported by 24 h dietary recall vs. 3-day food record.

Nutrient	n	r^2^	Fisher’s z’	*p*-Value
Energy	21	**0.78**	**1.38**	<0.00001
Carbohydrates	21	**0.84**	**1.56**	<0.00001
Lipids	21	**0.86**	**1.63**	<0.00001
Saturated Fats	21	**0.93**	**2.00**	<0.00001
Proteins	21	**0.87**	**1.68**	<0.00001
Fiber	21	**0.62**	**1.07**	0.00002
Sugar	21	**0.93**	**1.98**	<0.00001
Vitamin A	21	**0.37**	**0.70**	0.00366
Vitamin D	21	**0.93**	**1.99**	<0.00001
Vitamin E	21	**0.89**	**1.78**	<0.00001
Vitamin K	21	**0.87**	**1.67**	<0.00001
Sodium	21	**0.36**	**0.69**	0.00427
Calcium	21	**0.76**	**1.34**	<0.00001
Iron	21	**0.81**	**1.47**	<0.00001

Pearson’s r^2^ correlation coefficients and the corresponding Fisher’s z-transformations of the Pearson’s r^2^ correlation coefficients between caregiver-reported 24 h dietary recall vs. 3-day food records for energy, macronutrient, and micronutrient intake in individuals with ZSD. For heat map indications, red hues indicate a stronger correlation across assessment methods compared to blue hues, with darker red shades indicating stronger correlations compared to lighter red shades. Blue hues indicate a weak correlation between assessment methods, with darker blue shades indicating weaker correlations than lighter blue shades.

**Table 7:** Effect size of dietary assessment method for nutrient intake in individuals with ZSD.

Nutrient	n	24 h Recall Mean ± SD	Food Record Mean ± SD	Effect Size (±95% CI)	Hedges’ g
Energy	21	90.3 ± 12.8	90.0 ± 9.2	0.3 (−5.5, 6.1)	0.0
Carbohydrates	21	128.3 ± 18.1	122.0 ± 11.2	6.3 (−2.1, 14.7)	0.4
Proteins	21	262.7 ± 40.5	255.7 ± 27.1	7.0 (−7.3, 21.3)	0.2
Fat	21	154.2 ± 19.1	159.8 ± 8.3	−5.6 (−14.1, 2.9)	0.4
Saturated Fat	21	130.0 ± 20.3	135.8 ± 9.4	−5.8 (−15.5, 4.0)	0.4
Fiber	21	55.5 ± 12.3	52.8 ± 9.1	2.7 (−4.4, 9.8)	0.3
Sugar	21	214.2 ± 34.0	200.2 ± 30.2	14.0 (−0.7, 28.7)	0.4
Vitamin A	21	622.5 ± 218.2	590.4 ± 343.4	32.1 (−181.2, 245.4)	0.1
Vitamin D	21	241.2 ± 27.7	236.3 ± 35.4	4.9 (−8.6, 18.4)	0.2
Vitamin E	21	449.3 ± 67.7	415.7 ± 100.9	33.6 (11.9, 55.3)	0.4
Vitamin K	21	2624.8 ± 871.5	2909.0 ± 855.4	−284.2 (−781.3, 212.9)	0.3
Calcium	21	136.8 ± 30.0	128.0 ± 15.4	8.8 (−12.5, 30.1)	0.4
Iron	21	336.7 ± 131.7	191.4 ± 85.4	145.3 (105.6, 185.0)	1.3
Sodium	21	138.3 ± 35.6	362.8 ± 109.2	−224.5 (276.2, 172.8)	2.8

Effect size for dietary recall versus 3-day food record was evaluated for each energy and nutrient intake value by calculation of mean differences with 95% confidence intervals and assessment of Hedges’ g for magnitude of effect size. All but 3 nutrients (vitamin E, iron, and sodium) showed no significant effect size across dietary assessment methods.

## Data Availability

The raw data supporting the conclusions of this article will be made available by the authors on request.

## Supplementary Materials

The following supporting information can be downloaded at: https://www.mdpi.com/article/10.3390/nu17060989/s1, Figure S1: Correlation between dietary recall and 3-day food record of energy and macronutrient intake in subjects with ZSD; Figure S2: Correlation between dietary recall and 3-day food record of energy and macronutrient intake in subjects with ZSD.

## Abbreviations

The following abbreviations are used in this manuscript:

BMI	Body Mass Index
CDC	Centers for Disease Control
DHA	Docosohexaenoic acid
DRI	Dietary Reference Intake
GFPD	Global Foundation for Peroxisomal Disorders
kcal	Kilocalories
IOM	Institute of Medicine
NDSR	Nutrition Data System for Research
PBD	Peroxisomal biogenesis disorder
RCDP	Rhizomelic chondrodysplasia punctata
ZSD	Zellweger spectrum disorder
