# Supplementary material for: Comparison of Caregiver-Reported Dietary Intake Methods in Zellweger Spectrum Disorder
Source: Nutrients. 2025 Mar 12;17(6):989. doi: 10.3390/nu17060989 (PMC11944273; doi:10.3390/nu17060989)
Supplement: Supplementary file 1 [file nutrients-17-00989-s001.zip › nutrients-3465184-supplementary.pdf]

Supplementary Data

Supplementary Figure 1. Correlation between dietary recall and 3-day food record of energy and macronutrient intake in subjects with ZSD

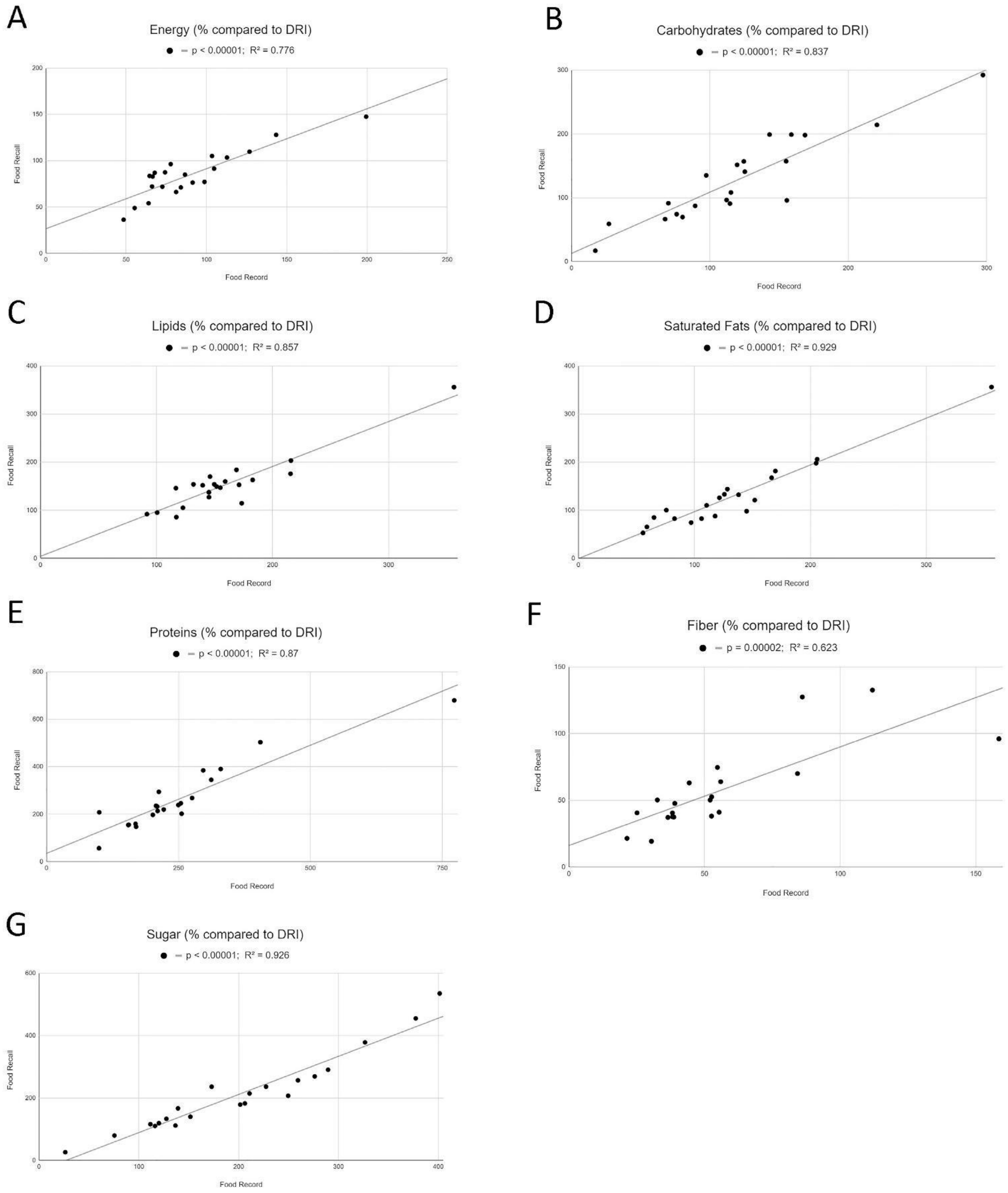

Supplementary Figure 1. Relationship between caregiver-reported 24-hour dietary recall *vs.* 3-day food record for energy intake (A), carbohydrate intake (B), total fat (lipid) intake (C), saturated fat intake (D), protein intake (E), fiber intake, and tota1 sugar intake (F). Data is reported as mean values for 3 dietary assessments (for both recall and food record) over a 6 month period ± standard deviation. Pearson's correlation coefficient for values is reported as r^2, with p-values <0.05 assigned as significant.

Supplementary Figure 2. Correlation between dietary recall and 3-day food record of micronutrient intake in subjects with ZSD.

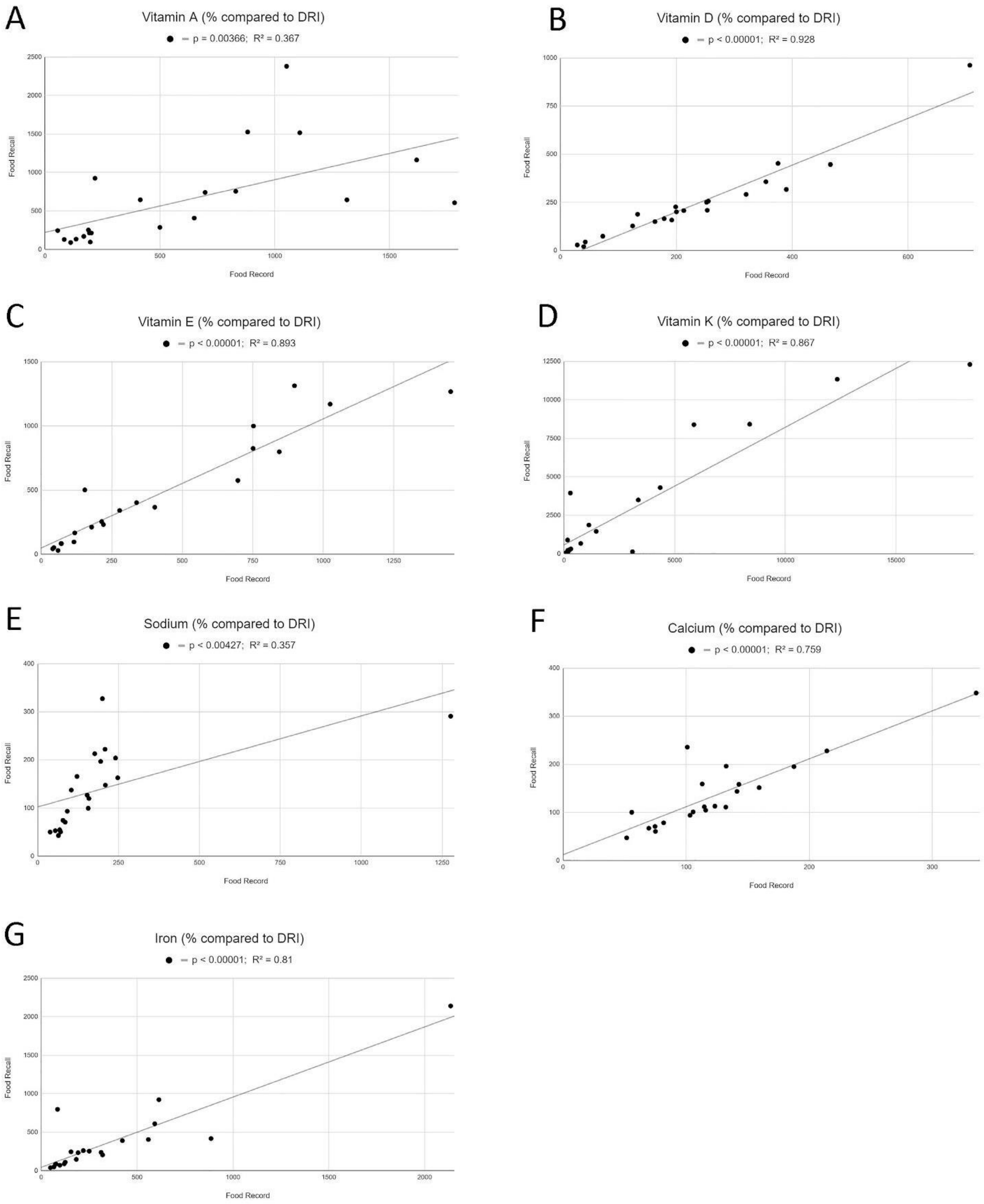

Supplementary Figure 2. Relationship between caregiver-reported 24-hour dietary recall *vs.* 3-day food record for Vitamin A intake (A), Vitamin D intake (B), Vitamin E intake (C), Vitamin K intake (D), sodium intake (E), calcium intake, and iron intake (F). Data is reported as mean values for 3 dietary assessments (for both recall and food record) over a 6 month period ± standard deviation. Pearson's correlation coefficient for values is reported as r^2, with p-values <0.05 assigned as significant.
